# Effect of Alcoholic Extracts of Indian Medicinal Plants on the Altered Enzymatic Activities of Diabetic Rats

**DOI:** 10.4103/0250-474X.58175

**Published:** 2009

**Authors:** E. N. Sundaram, P. Uma Maheswara Reddy, K. P. Singh

**Affiliations:** Drug Standardisation Unit (H), O. U. B. No. 32, Street No. 4, Vikrampuri, Habsiguda, Hyderabad-500 007, India; 1 Department of Zoology, Osmania University, Hyderabad-500 007, India

**Keywords:** STZ-induced diabetes, alcoholic extracts of medicinal plants, SGOT and SGPT activities, serum urea concentration and serum total protein and albumin concentration

## Abstract

In present study, the effect of alcoholic extract of *Momordica charantia, Aegle marmelos* and *Eugenia jambolana* was studied on serum glutamic oxaloacetate transminase and serum glutamic pyruvate transminase activities and on serum urea, total protein and albumin concentrations of streptozotocin diabetic rats. Diabetes in rats was induced by single dose of streptozotocin (30 mg/kg i. p.). On confirming the diabetes after 48 h of injection, alcoholic extracts of three plants were administered orally in doses of 250 mg and 500 mg/kg/d for 30 d. Glibenclamide (300 μg/kg/d) was used as a reference drug for comparison. Streptozotocin diabetic rats showed a significant increase in serum glutamic oxaloacetate transminase and serum glutamic pyruvate transminase activities and serum urea concentration but a significant decrease in serum total protein and albumin concentrations and albumin/globulin ratio. Oral administration of alcoholic extract of *Momordica charantia, Aegle marmelos* and *Eugenia jambolana* in daily doses of 250 mg and 500 mg/kg for a period of 1 mo produced dose- and duration-dependent decrease in serum glutamic oxaloacetate transminase and serum glutamic pyruvate transminase activities as well as decrease in serum urea concentration and restored the serum total protein and albumin concentration and albumin/globulin ratio to a great extent in streptozotocin diabetic rats. The beneficial effects of these plants in 500 mg/kg dose in streptozotocin diabetic rats were comparable to that of glibenclamide (300 μg/kg), a standard oral hypoglycaemic drug used in clinical practice.

Impaired utilization of glucose due to lack of insulin secretion or its action has been reported to alter the enzymatic activities in diabetic patients as well as in experimental animals[1–3]. Diabetic animals also showed an increased break down of muscles and other tissues proteins into amino acids due to enhanced proteases activity which in turn resulted into increased urea levels in the blood[4]. Likewise, increased activity of serum glutamic oxaloacetate transminase (SGOT) and of serum glutamic pyruvate transminase (SGPT) in diabetes is of clinical importance[2] because elevated activity of SGOT is suggestive of cardiac damage and that of SGPT liver damage[3].

At present, the pathophysiology of diabetes mellitus is most precisely understood. Being multiple defect disease, it is not worthwhile to prescribe a drug that selectively targeted a single defect i.e. only to control blood glucose concentrations within the normal limit since such regimen may not be of much help in alleviating late clinical manifestations of diabetes mellitus. On the other hand, the multiple activities of plant based medicinal preparations offer an enormous scope for not only to control the hyperglycaemia of diabetes mellitus but also its late complications. Moreover, WHO study groups have emphasized strongly the optimal rationale uses of traditional medicines because the theory of polyherbal formulations having synergistic, potentiative, agonistic, antagonistic pharmacological actions within themselves work together in a dynamic way[5]. For last several decades, many Indian scientists have studied the antidiabetic effect of medicinal plants together with their beneficial effects in complications associated with diabetes[6,7].

In order to study the spectrum of effectiveness other than hypoglycaemic activity of *M. charantia, A. marmelos* and *E. jambolana,* the present study was undertaken to find out the effectiveness of alcoholic extracts of these plants on the altered enzymatic activities responsible for cardiac and liver damage and protein catabolism of diabetic rats.

Wistar rats of both sexes (175-200 g) procured from National Centre for Laboratory Animal Sciences, National Institute for Nutrition, Hyderabad, India, housed (12/12 h, light/dark cycles, room temp. 21-23°) in polypropylene cages (47×34×20 cm) were acclimatized to the laboratory conditions for 10 d before use. They were fed nutritious pelleted diet and water *ad libitum* through out the study.

Streptozotocin, STZ (Sigma, St Louis, USA) for induction of diabetes, glibenclamide (Aventis Pharma, Mumbai, India), a standard oral hypoglycaemic drug, readymade kits/ reagents for estimation of SGOT and SGPT (Medsource Ozone Biochemicals, Faridabad, India), urea (Excel Diagnostic, Hyderabad, India) and for total protein and albumin (Span Diagnostics, Surat, India) were used. Concentrated lyophilised alcoholic extract of *M. charantia,* and *A. marmelos* fruits, and *E. jambolana* seeds were dissolved in distilled water by stirring in a concentration of 50 mg/ml before use.

Experimental protocol was approved by Institutional Animals Ethics Committee. Diabetes in rats was induced by a single dose of STZ (30 mg/kg i. p.). On confirming the diabetes after 48 h of injection, rats were divided into 9 groups of 6 each. i) nondiabetic control, ii) STZ diabetic control given normal saline, iii and iv) STZ diabetic rats given 250 mg and 500 mg/kg/d of *M. charantia* fruit extract, v and vi) STZ diabetic rats given 250 mg and 500 mg/kg/d of *A. marmelos fruit* extract, vii and viii) *STZ* diabetic rats given 250 mg and 500 mg/kg/d of *E. jambolana* seeds extract and ix) STZ diabetic rats given 300 μg/kg/d of glibenclamide for 30 d, orally[8].

SGOT and SGPT activities (Modified Reitman and Frankel's method)[9,10], and concentrations of urea (Berthelot method)[11], total protein (Biuret method)[12] and albumin (Bromocresol green method)[13] in the serum of diabetic rats were measured. For SGOT and SGPT activities, absorbance of serum samples, standard, calibrator, and blank were measured at 505 nm against distilled water while absorbance of serum samples and standard for urea at 570 nm, for total protein at 578 nm and for albumin at 630 nm were measured with their respective blanks on a spectrophotometer (Systronics, Ahmedabad, India). Mean values with standard error of the mean of each group were calculated. The level of significance of difference (p<0.05) between diabetic and nondiabetic groups and between alcoholic extract/glibenclamide and diabetic groups were analysed using the student's - t test[14].

There was a progressive increase in SGOT and SGPT activities of STZ diabetic control rats. The increase in enzymatic activities was significant even after 12 d of STZ injection. At the end of 1 mo, SGOT and SGPT activities of diabetic rats increased by 36.2% and 66.7%, respectively when compared to that of nondiabetics (Table 1). On the other hand, oral administration of alcoholic extracts of *M. charantia, A. marmelos* and *E. jambolana* showed dose- and duration-dependent decrease in SGOT and SGPT activities in STZ diabetic rats. At the end of 1 mo, SGOT activity decreased by 19.8% and 24.5% and SGPT activity by 34.5% and 50.0% with 250 mg and 500 mg/kg/d doses of *M. charantia*, respectively. Likewise, *A. marmelos* decreased SGOT activity by 12.4% and 24.9% and SGPT activity by 21.0% and 46.9% while *E. jambolana* decreased SGOT activity by 12.4% and 19.3% and SGPT activity by 22.6% and 44.0% respectively in 250 mg and 500 mg/kg/d doses. Glibenclamide (300 μg/kg) also decreased SGOT and SGPT activities by 27.5% and 46.4%, respectively (Table 1).

**TABLE 1:** EFFECT OF ALCOHOLIC EXTRACTS OF *M. CHARANTIA, A. MARMELOS* AND *E. JAMBOLANA* ON SGOT AND SGPT ACTIVITIES OF STZ DIABETIC RATS AFTER 1 MO OF TREATMENT.

Groups	SGOT activity		SGPT activity	
		
	mU/ml	Percent change (+/−)@	mU/ml	Percent change (+/−)@
Nondiabetic	28.5±2.95	-	25.2±2.42	-
Diabetic	38.8±2.67*	36.2 (+)	42.0±4.65*	66.7 (+)
*M. charantia*				
250 mg	31.2±3.07^NS^	19.8 (−)	27.5±3.52^a^	34.5 (−)
500 mg	29.3±1.62^a^	24.5 (−)	21.0±3.13^a^	50.0 (−)
*A. marmelos*				
250 mg	34.0±3.45^NS^	12.4 (−)	33.2±3.41^NS^	21.0 (−)
500 mg	29.2±1.47^a^	24.9 (−)	22.3±2.64^a^	46.9 (−)
*E. jambolana*				
250 mg	34.0±3.54^NS^	12.4 (−)	32.5±2.91^NS^	22.6 (−)
500 mg	31.3±1.58^a^	19.3 (−)	23.5±2.39^a^	44.0 (−)
Glibenclamide				
300 μg	28.2±1.76^a^	27.5 (−)	22.5±2.29^a^	46.4 (−)

Significantly different

* (≤0.05) from nondiabetic control, Significantly different

a (≤0.05) from diabetic control

NS - not significant

@ Percent change from (+) nondiabetic control and from (−) diabetic control, n= 6 is the number of animals used in each group.

STZ diabetic rats showed significant increase in urea concentration in serum even after 12 d of STZ injection. After 1 mo period, the percent increase in serum urea concentration was of the order at 58.8% when compared with that of nondiabetic rats (Table 2). Very similar to the effect on SGOT and SGPT activities, alcoholic extracts of *M. charantia, A. marmelos* and *E. jambolana* showed dose- and duration-dependent decrease in serum urea concentration of STZ diabetic rats. At the end of 1 mo, the percent decrease in serum urea concentration with 250 mg and 500 mg/kg/d doses was 27.2% and 33.2% with *M. charantia*, 25.2% and 30.0% with *A. marmelos* and 22.9% and 25.6% with *E. jambolana* extracts. Glibenclamide (300 μg/kg) also decreased serum urea concentration by 30.6% during the same period (Table 2).

**TABLE 2:** EFFECT OF ALCOHOLIC EXTRACTS OF *M. CHARANTIA, A. MARMELOS* AND *E. JAMBOLANA* ON SERUM UREA CONCENTRATION OF STZ DIABETIC RATS AFTER 1 MO OF TREATMENT

Groups	Urea concentration	
	
	mg/100 ml	Percent change (+/−)@
Nondiabetic	31.3±2.20	-
Diabetic	49.7±1.76*	58.8 (+)
*M. charantia*		
250 mg	36.2±2.52^a^	27.2 (−)
500 mg	33.2±2.86^a^	33.2 (−)
*A. marmelos*		
250 mg	37.2±4.21^a^	25.2 (−)
500 mg	34.8±3.12^a^	30.0 (−)
*E. jambolana*		
250 mg	38.3±3.14^a^	22.9 (−)
500 mg	37.0±2.56^a^	25.6 (−)
Glibenclamide		
300 μg	34.5±1.91^a^	30.6 (−)

Significantly different

* (≤0.05) from nondiabetic control, Significantly different

a (≤0.05) from diabetic control

@ Percent change from (+) nondiabetic control and from (−) diabetic control, n=6 is the number of animals used in each group.

Table 3 shows that there was a significant decrease in serum total protein and albumin concentrations of STZ diabetic rats when compared to that of nondiabetics. The decrease in total protein and albumin concentrations in the serum of diabetic rats was significant even after 12 d of STZ injection. At the end of 1 mo, the percent decrease observed in total protein and albumin concentrations in the serum of diabetic rats was 23.5% and 33.4%, respectively.

**TABLE 3:** EFFECT OF ALCOHOLIC EXTRACTS OF *M. CHARANTIA, A. MARMELOS* AND *E. JAMBOLANA* ON SERUM TOTAL PROTEIN AND ALBUMIN CONCENTRATIONS AND ON ALBUMIN/ GLOBULIN RATIO OF STZ DIABETIC RATS AFTER 1 MO OF TREATMENT.

Groups	Serum total protein		Serum albumin		Albumin/globulin ratio
			
	g/100 ml	Percent change (+/−)@	g/100 ml	Percent change (+/−)@
Nondiabetic	6.97±0.31	-	4.13±0.26	-	1.46
Diabetic	5.33±0.37*	23.5 (−)	2.75±0.13*	33.4 (−)	1.00
*M. charantia*					
250 mg	6.77±0.23^a^	27.0 (+)	3.70±0.23^a^	34.5 (+)	1.22
500 mg	7.02±0.21^a^	31.7 (+)	3.88±0.17^a^	41.1 (+)	1.19
*A. marmelos*					
250 mg	6.23±0.17^a^	16.9 (+)	3.37±0.15^a^	22.5 (+)	1.18
500 mg	6.65±0.27^a^	24.8 (+)	3.60±0.09^a^	30.9 (+)	1.18
*E. jambolana*					
250 mg	6.38±0.38^a^	19.7 (+)	3.45±0.09^a^	25.5 (+)	1.19
500 mg	6.68±0.16^a^	25.3 (+)	3.08±0.12^a^	33.8 (+)	1.23
Glibenclamide					
300 μg	6.65±0.24^a^	24.8 (+)	3.73±0.29^a^	35.6 (+)	1.28

Significantly different

* (≤0.05) from nondiabetic control, Significantly different

a (≤0.05) from diabetic control

@ Percent change from (+) nondiabetic control and from (−) diabetic control, n=6 is the number of animals used in each group.

Alcoholic extracts of *M. charantia, A. marmelos* and *E. jambolana* produced dose- and duration-dependent increase in serum total protein and albumin concentrations of STZ diabetic rats. The effect became significant only after 20 d of treatment. After 1 mo, the percent increase in total protein concentration of STZ diabetic rats in doses of 250 mg and 500 mg/kg of *M. charantia* was 27.0% and 31.7%, with *A. marmelos* 16.9% and 24.8% and that of *E. jambolana* was 19.7% and 25.3% respectively, whereas, the percent increase in serum albumin concentration of STZ diabetic rats with 250 mg and 500 mg/kg doses of *M. charantia* was 34.5% and 41.1%, of *A. marmelos* 22.5% and 30.9% and that of *E. jambolana* was 25.5% and 33.8% respectively. Glibenclamide (300 μg/kg) also increased total protein and albumin concentrations in the serum of STZ diabetic rats by 24.8% and 35.6%. Table 3 also shows an improvement in albumin/globulin ratio in STZ diabetic rats treated with three plants extracts and glibenclamide when compared with that of diabetic controls.

In diabetic patients, altered enzymatic activity of SGOT and SGPT is of clinical importance[2]. The elevated enzymatic activity of SGOT with only moderately increase in SGPT activity suggests cardiac damage while elevated activity of SGPT with only moderate increase in SGOT suggests liver damage[3]. In the present study also, there was a significant increase in SGOT (36.2%) and SGPT (66.7%) activities of STZ diabetic rats when compared with that of nondiabetic rats. Alcoholic extracts of *M. charantia, A. marmelos* and *E. jambolana* showed a progressive dose- and duration-dependent decrease in SGOT and SGPT activities which was significant only with 500 mg/kg doses after 30 d of treatment. Similar depressant effect on SGOT and SGPT activities of STZ diabetic rats was also observed with glibenclamide (300 μg/kg). The results of the present study are in agreement with those of Sivajothi *et al*.[15]. The order of effectiveness in depressing enzymatic activity was glibenclamide>*A. marmelos*>*M. charantia*>*E. jambolana* for SGOT and *M. charantia*>*A. marmelos*>glibenclamide>*E. jambolana* for SGPT activities. The better response of *M. charantia* and *A. marmelos* on SGPT activity than that of glibenclamide clearly indicates that both plants have better hepatic protective effect as compared to that of glibenclamide.

Diabetic animals manifest negative nitrogen balance due to enhanced activity of proteases enzymes responsible for breakdown of proteins into amino acids in the muscles and other tissues leading to increased production of urea in the body[4]. In addition, degenerative changes observed in the tubular epithelium of renal tubules of diabetic animals also contribute to the rise in serum urea concentrations due to impairment of urea excretion by kidneys[16]. In the present study, serum urea concentration of STZ diabetic rats increased by 58.8% (p-<0.05) when compared to that of nondiabetic rats. On the contrary, alcoholic extracts of three plants showed a progressive dose- and duration-dependent decrease in serum urea concentration of STZ diabetic rats. The percent decrease observed in the serum urea concentration of STZ diabetic rats at the end of 1 mo with daily doses of 500 mg/kg of *M. charantia, A. marmelos* and *E. jambolana* was comparable to that of glibenclamide (300 μg/kg). Present results are in agreement with the findings of earlier workers[17,18]. The order of effectiveness for decreasing serum urea concentration of STZ diabetic rat was *M. charantia>Glibenclamide≥A. marmelos>E. jambolana.*

On the contrary, STZ diabetic rats showed a significant decrease in the serum total protein and albumin concentrations and albumin/globulin ratio. The percent decrease observed in the total protein and albumin concentrations at the end of 1 mo study was 23.5% and 33.4%, respectively. Similar depressant effect on serum total protein and albumin concentrations in STZ diabetic rats has also been reported by some workers[15,19,20].

Restoration of serum total protein and albumin concentrations and albumin/globulin ratio of STZ diabetic rats with alcoholic extracts of these plants were dose-and duration-dependent. On last d of study, *M. charantia, A. marmelos* and *E. jambolana* at doses of 500 mg/kg increased total protein concentration by 31.7%, 24.8% and 25.3% and serum albumin concentration by 41.1%, 30.9% and 33.8%, respectively very similar to that of glibenclamide (300 μg/kg). Earlier, Dighe *et al*[21] had reported that accelerated proteolysis of uncontrolled diabetes occurs as a result of deranged glucagons-mediated regulation of cyclic AMP formation in insulin deficiency. In addition, secondary hypoalbumenia commonly observed in diabetic patients is generally attributed with the nephrotoxicity[22]. The restoration of total protein and albumin concentrations and albumin/globulin ratio in the serum of STZ diabetic rats by three alcoholic extracts and glibenclamide may be due to inhibition of proteolytic activity due to enhance insulin secretion and proper utilization of blood glucose. Similar effects have also been reported by some workers[15,18] on total protein and albumin concentrations. The order of effectiveness in restoring serum total protein was *M. charantia>E. jambolana>A. marmelos>* glibenclamide and for serum albumin was *M. charantia>glibenclamide>E. jambolana>A. marmelos*. Thus, it is concluded that alcoholic extracts of *M. charantia, A. marmelos* and *E. jambolana* appears to be better alternative in restoring altered enzymatic activity of diabetic patients as these medicinal plants are devoid of any untoward/toxic effects.
